# Overview of approved and upcoming vaccines for SARS-CoV-2: a living review

**DOI:** 10.1093/oxfimm/iqab010

**Published:** 2021-05-22

**Authors:** Jennifer Alderson, Vicky Batchelor, Miriam O’Hanlon, Liliana Cifuentes, The Oxford-Cardiff COVID-19 Literature Consortium, Felix Clemens Richter, Jakub Kopycinski

**Affiliations:** 1 Kennedy Institute of Rheumatology, Nuffield Department of Orthopaedics, Rheumatology and Musculoskeletal Sciences, University of Oxford, Oxford, United Kingdom; 2 Nuffield Department of Medicine, University of Oxford, Oxford, United Kingdom

**Keywords:** SARS-CoV-2, Vaccine, Variant, Efficacy, Effectiveness

## Abstract

The rapid design and implementation of SARS-CoV-2 vaccines is testament to a successfully coordinated global research effort. While employing a variety of different technologies, some of which have been used for the first time, all approved vaccines demonstrate high levels of efficacy with excellent safety profiles. Despite this, there remains an urgent global demand for COVID-19 vaccines that requires further candidates to pass phase 3 clinical trials. In the expectation of SARS-CoV-2 becoming endemic, researchers are looking to adjust the vaccine constructs to tackle emerging variants. In this review, we outline different platforms used for approved vaccines and summarise latest research data with regards to immunogenicity, dosing regimens and efficiency against emerging variants.

## Introduction

The betacoronavirus, severe acute respiratory syndrome coronavirus 2 (SARS-CoV-2) has led to the most challenging global health crisis in the last hundred years. Infection can manifest itself in a range of clinical symptoms, which together are defined as coronavirus disease 2019 (COVID-19). COVID-19 has so far claimed over 2.6 million lives worldwide ($17^{th}$ March 2021) and led to long-term health impacts in many more. Compounding the medical implications of the pandemic, the associated economic, social and mental health burden will likely take years to resolve.As a member of the coronoviridae family, SARS-CoV-2 contains a positive-sense, single-stranded RNA genome which, along with other structural and non-structural proteins (extensively reviewed elsewhere (Arya et al., 2021)), encodes a spike glycoprotein composed of a trimer of S1 and S2 heterodimers. The S1 component contains a receptor binding domain (RBD) that allows infection of mammalian cells via the angiotensin-converting enzyme 2 (ACE-2) receptor (Walls et al., 2020). Binding to ACE-2 induces a conformational change in the S glycoprotein, allowing the S2 component to mediate fusion with the host cell membrane (Huang, Yang, Xu, Xu, & Liu, 2020; Walls et al., 2020). Upon entry into the cell proteins encoded by virus are directly translated using host ribosomes while the viral genome is replicated by the viral replicase. Viral progeny exit the host cell on assembly, with nascent virus particles expelled from the respiratory tract by coughing or breathing, within droplets or as aerosols.

Because of its immunodominance and critical role in cell entry, the spike protein encoded either by DNA within a viral vector or mRNA, or produced as a recombinant adjuvanted protein, has been the focus for most vaccine efforts. In the majority of vaccine candidates, the spike protein sequence has been modified through the incorporation of paired proline residues. This modification has been found to stabilise the S protein in its prefusion conformation, which simultaneously increases its levels of expression and antigenicity. (Sanders et al 2021)

In this living review, we describe different strategies employed in SARS-CoV-2 vaccine development, ranging from the use of established technologies such as inactivated or subunit vaccines, to novel platforms such as lipid-encapsulated mRNA. Taking together all approved vaccines and current phase 3 clinical trial vaccine candidates, we will summarise the most recent published data on their efficacy and immunogenicity and discuss future research directions.

## Inactivated Vaccines

This technology, previously used in the Salk Polio and rabies vaccines (HDBC and CCEEV), uses susceptible cell lines to propagate live virus, which can subsequently be harvested, purified, and finally inactivated. Inactivated vaccines differ from attenuated virus vaccines as, despite retaining all viral structural antigens, the virus itself is unable to replicate. As such, inactivated vaccines have improved safety profiles and demonstrate excellent stability without the requirement for -80$^{o}$C storage, thus facilitating vaccine distribution. However, they are often weakly immunogenic and require adjuvants and boosting. While historically formalin was used to inactivate pathogens, it has been known to damage or alter the antigenic properties of proteins potentially leading to aberrant immune responses (Killikelly, Kanekiyo, & Graham, 2016). This risk has been mitigated by introduction of β-propiolactone as an inactivating agent.

At present, two inactivated vaccine candidates for SARS-CoV-2 are in development (Table 1): CoronaVac (Sinovac, ClinicalTrials.gov NCT04352608) and BBIB-CorV (Sinopharm, ClinicalTrials.gov NCT04352608.). Both use plaque-purified isolates propagated in Vero cells (Xia et al., 2021; Zhang et al., 2021), BBIB-CorV is subsequently inactivated by β-propiolactone, while CoronaVac is inactivated using a combination of β-propiolactone and formaldehyde. Both vaccines are administered intramuscularly (IM) with an aluminium hydroxide adjuvant (Palacios et al., 2020).

So far, Sinopharm's BBIB-CorV, derived from the HB02 strain, is the only inactivated vaccine in human clinical trials that has been tested on participants above 60 years of age. Phase 1 dose-escalation studies assessing prime and boosting at 28 days with 2, 4 and 8 µg doses, showed all doses to be well-tolerated, with fever, pain at the vaccination site, and fatigue being the most common side-effects. While inducing seroconversion in 100% of individuals at lowest dose (2µg), seroconversion was delayed until day 42 in the >60 age group, compared with day 28 in the younger 18-59 group. Although neutralising antibodies (nAbs) were detected in all vaccinees across dose groups by day 42 post-boost, titres were also found to be lower in the >60 group. In the follow up phase 2 study, while inducing lower antibody titres than two dose 2 and 4µg regimens, a single 8µg dose was found to induce seroconversion in 75% of individuals in the 18-59 age group (Xia et al., 2021). This vaccine, described as having 79% efficacy by Sinopharm, without publication of phase 3 trial data, was initially approved in China, UAE, Bahrain prior to more recent approval in other countries. Phase 3 data is likely to include more detailed information on immunogenicity, which had not previously been reported in phase 1 and 2. The CoronaVac vaccine, derived from the CN02 isolate, has already been licensed for use in China. Phase 1 and 2 trials assessed the vaccine in 144 and 600 individuals respectively (ages 18-59). The vaccine was well-tolerated, with common side effects including pain at the injection site and fatigue. T Cells measured by ELISpot reported in phase 1, had a maximum high average of 7.4 SFC/100,000 cells, 14 days post boost, across all groups. (https://doi.org/10.1016/ S1473-3099(20)30831-8)

Phase 2 studies measured responses to two doses, with boosting at either day 14 or 28. Highest rates of seroconversion were found at 28 days post-boost. Although noting that titres of nAbs were lower than those found in convalescent patients, the study authors suggest that memory B-cells may play a more important role in preventing infection than nAb titres, citing SARS and MERS as examples of viruses where reinfections are rare despite waning nAb titres (Zhang et al., 2021). Estimates of efficacy in phase 3 clinical trials in Brazil, Indonesia and Turkey were 50.4%, 65.3% and 91.25% respectively. This wide range is likely due both to differences in trial methodology, and the l number of participants included in efficacy analysis.

Phase 3 trial of CoronaVac (NCT04651790) which should have efficacy data for people above 60 s expected to be completed by January 2022.

***RNA Vaccines***

To date, two mRNA vaccines have been approved for use following the successful completion of phase 3 efficacy trials (Table 2). Both vaccines encode the stabilised prefusion form of the SARS-CoV-2 spike glycoprotein trimer derived from the MN908947.3 sequence. This sequence is flanked by a 5' CAP and an untranslated region (UTR), with a further UTR and poly-A-tail at the 3' end. In both, the RNA has been modified to contain pseudouridine in place of uracil; this allows the evasion of host innate immune responses, which increases the efficiency of translation of the vaccine transgene (Mulligan et al., 2020). The mRNA is subsequently encapsulated in a PEGylated lipid nanoparticle, which facilitates cell entry of the administered vaccine.

RNA vaccines have several advantages over other vaccine strategies: they are non-infectious, cannot integrate into the host genome and do not induce vector-specific responses (Pardi, Hogan, Porter, & Weissman, 2018). Also as synthetic molecules, the can be quickly adapted to new emerging variants which is of particular interest with SARS-CoV-2. Furthermore, single and double stranded RNA moieties can prime various facets of innate immune responses through recognition by endosomal proteins such as Toll-like receptors (TLR3 and TLR7), and components of the inflammasome in the cytosol (Teijaro & Farber, 2021).

The Pfizer/BioNTech vaccine was the first SARS-CoV-2 vaccine to be approved by the UK's Medicines and Healthcare Products Agency (MHRA) in December 2020. Initial phase 1 dose-escalation studies

(Walsh et al., 2020) were performed to compare the safety and immunogenicity of secreted (BNT162b1) and membrane-bound (BNT162b2) forms of the glycoprotein. A two-dose regimen assessing 10µg, 20µg and 30µg doses was performed in parallel with a single dose regimen of 100µg. Although both vaccine candidates induced similar dose-dependent immunogenicity profiles, boosting significantly improved immunogenicity, while BNT162b2 was found to be associated with lower incidence and severity of systemic reactions.

In a randomised, placebo-controlled phase 3 trial (NCT04368728 Polack et al., 2020), two 30µg doses were given IM to 21,720 individuals 21 days apart, with a matched saline control given to the 21,728 volunteers in the placebo group. The primary outcome was COVID-19 disease measured 7 days following the second dose. Data showed disease in 8 of 18,198 vaccinated individuals with no prior evidence of infection, compared with 162 of 18,325 in the placebo group. Geometric mean titres (GMT) of vaccine-induced neutralising antibodies (nAbs) were compared with sera isolated from convalescent donors. At 7-14 days post-boost, GMTs were between 1.7 and 4.6x greater than the GMT of convalescent sera in young adults (aged 18-55), and 1.6x greater in older adults (aged 65-85). Müller et al. (2021) assessed levels of nAbs induced in a cohort of individuals aged <60 and >80 that had received two doses of BNT162b2 and found a reduction in nAb levels in the older cohort. Furthermore, 31.3% of the >80-year-olds had no detectable antibodies compared with only 2.2% in the <60year group, a possible consequence of age-related immune senescence. This may be related to the decreased potential of older individuals to mount an effective immune memory response to vaccines. While T cell responses were not directly measured to BNT162b2, the BNT162b1 candidate was shown capable of inducing both Th1-polarised CD4 and cytotoxic CD8 responses (Sahin et al., 2020).

The mRNA-1273 vaccine, co-developed by Moderna Inc. and the NIAID, was first given emergency use authorisation by the FDA on December 18th, 2020. Phase I dose-escalation and safety studies showed the vaccine to be well-tolerated with GMT of neutralising antibodies in volunteers receiving 100 or 200µg dose having a similar distribution to those from convalescent patients. Th1-polarised T-cell responses, which are important for the control of intracellular pathogens such as viruses, were also induced with some low frequency CD8 responses. However, increases in the frequency of adverse events was seen at the highest dose, particularly following boost (Jackson et al., 2020).

In a double-blinded, placebo-controlled efficacy phase 3 COVE trial (NCT04470427), 14,134 individuals were given 100µg of the mRNA-1273 vaccine as IM homologous prime boost (1-month interval), while 14,073 received a placebo (Baden et al., 2020). The primary endpoint was the prevention of COVID-19 illness onset at least 14 days post-boost. Following unblinding, symptomatic COVID-19 illness was found in 185 and 11 participants in the placebo and vaccine arms, respectively. When stratified by age, the efficacy was 95.6% in those aged 18-65, and 86.4% in individuals above 65 year.

A further nanoparticle-enveloped, mRNA vaccine has been developed by CureVac; initial phase 1 prime-boost, dose-escalation studies assessed the safety and immunogenicity of a 2 to 12 μg dose range in two age groups,18-40 and 41-60 (Kremsner et al., 2020) following a 28 day interval. Results showed seroconversion in all vaccinees 12 days post-boost with neutralising antibody titres comparable with those found in the serum of convalescent patients.

## ***Protein vaccines***

Subunit vaccines are produced as recombinant antigens using bacterial, yeast or insect expression systems. They can be subdivided into two groups: protein subunit and self-assembling virus-like particle (VLP) vaccines; production is readily scalable, and subunit vaccines exhibit both excellent stability and safety profiles. While antigens are selected based on their capacity to elicit humoral responses, protein vaccines seldom induce strong immune responses and often require adjuvanted booster vaccinations (Vartak & Sucheck, 2016). Poor immunogenicity may result from an inability to induce an immune response through stimulation of pattern recognition receptors (PRR), the short half-life of exogenous proteins in the body, and finally whether the expression system enables post-translational modification (e.g. glycosylation) of the antigen. This vaccine platform has previously been successfully employed for the hepatitis B virus surface antigen (HBsAg) and human papillomavirus vaccines (Nascimento & Leite, 2012). Preliminary results are only available from one protein vaccine candidate in phase 3 trials so far, this section will briefly discuss the ongoing research for promising protein subunit vaccines currently in development.

NVX-CoV2373 (Table 3) is a recombinant baculovirus-expressed nanoparticle vaccine adjuvanted with Matrix-M1-adjuvant. A randomised, placebo-controlled phase 1-2 clinical trial using a two-dose regimen, given IM following a 21 day interval, showed a positive correlation between nAb concentrations and anti-spike IgG (C. Keech et al., 2020; Cheryl Keech et al., 2020). The vaccine demonstrated a good safety profile with only mild adverse events reported and no serious adverse events. The Matrix-M1 adjuvant combined with 5μg of nanoparticles induced anti-S IgG levels equivalent to 25μg nanoparticles alone. Furthermore, vaccinees given the adjuvant with both 5μg and 25μg nanoparticles induced nAbs 100-fold higher than those given 25μg nanoparticles alone. Recently, Novavax announced that efficacy for the NVX-CoV2373/Matrix-M1 vaccine a phase 3 trial (NCT04611802) was 96.4% in preventing mild, moderate and severe disease in the UK , this reduced to55.4% in South Africa, where the majority of cases were due to B1.351 variant. Most importantly, the vaccine attained 100% efficacy against severe disease, including all hospitalisation and death (Novavax, March 11 2021).

ZF2001 is the second recombinant protein vaccine to have started phase 3 clinical trials. This vaccine encodes the RBD antigen and is adjuvanted with aluminium hydroxide. Although there is a lack of preclinical data on the vaccine, the results of both phase 1 and phase 2 trials, consisting of 50 and 900 healthy adults respectively, are published as a preprint (Yang et al., 2020). There were no severe adverse reactions to the vaccine, with only mild side-effects being reported, all of which resolved within four days of inoculation. However, in the phase 2 trial seven participants reported serious adverse events which the investigators inferred to be unrelated to the vaccine. Individuals in the phase 1 clinical trial received three IM doses of the adjuvanted RBD-dimer vaccine, 30 days apart, with the placebo group receiving adjuvant alone. The phase 2 clinical trial included groups receiving either two or three doses of 25μg or 50μg. Two weeks after the final dose, the two-dose group demonstrated seroconversion rates of 76% (at 25μg) and 72% (at 50μg). In comparison, groups receiving three doses showed an increase to 97% (at 25μg) and 93% (at 50μg) seroconversion at the same time point. As there was no evidence for a dose-dependent enhancement in immunogenicity, the phase 3 clinical trial is proceeding with three-doses of 25μg. The trial is expected to conclude by April 2022 ("A Phase III Clinical Trial to Determine the Safety and Efficacy of ZF2001 for Prevention of COVID-19,").

***Viral Vectored Vaccines***

Viral vectored vaccines employ recombinant viruses modified to encode antigens derived from the target pathogen to directly infect host cells. Upon infection, the host cell produces and displays the antigen of interest, triggering an immune response. Viral vectored platforms have previously been used against several infectious disease, the most notable being a recombinant vesicular stomatitis virus vaccine against Ebola (rVSV-ZEBOV), which was developed in response to the 2013 outbreak (Henao-Restrepo et al., 2015). Prior to the COVID-19 pandemic, no adenovirus-based vaccine had been licensed for use in humans, with pre-existing immunity to circulating adenoviruses suggested as a potential barrier for their use (Tatsis & Ertl, 2004).

Four viral-vectored SARS-CoV-2 vaccine candidates have reached phase 3 clinical trials (Table 4). All are replication-deficient adenoviruses expressing the SARS-CoV-2 spike protein (Folegatti et al., 2020; Denis Y. Logunov et al., 2020; Sadoff et al., 2020; Zhu, Li, et al., 2020) and have previously been extensively characterised as viral vectors.

Phase 1 and 2 trials, testing dose ranges between 5 x $10^9$ and 1.5 x $10^{11}$ viral particles (vp), showed minor side-effects common to all adenoviral vector candidates: primarily pain at site of injection, headache, and fever (Folegatti et al., 2020; Denis Y. Logunov et al., 2020; Sadoff et al., 2020; Zhu, Li, et al., 2020). Although Ad5-nCoV demonstrated similar immunogenicity across doses, side effects occurred at increased frequencies at the highest dose (Zhu, Guan, et al., 2020). While AstraZeneca found all 3 doses to be well-tolerated, increased doses were concomitant with nAb levels (Folegatti et al., 2020). Both Ad26.COV2.S and Ad5-nCoV tested a single-dose regimen in phase 3 trials.

Despite methodological variation between studies, all candidates were found to induce both humoral and cellular responses. Levels of anti-RBD or anti-spike IgG peaked at day 28, while T cell responses, measured by IFN-γ ELISpot, peaked at day 14 post-boost. All studies found a positive correlation between antibody titres against the RBD or S and the nAb titres. Furthermore, study authors noted that CD4+ T cell responses induced by Ad26.COV2.S and ChAdOx1 nCoV-19 were Th1-biased (Ewer et al., 2020; Sadoff et al., 2020).

Initial analysis of the vaccine efficacy for ChAdOx1 nCoV-19 were based on a meta-analysis of two smaller single-blind clinical trials (VoyseyClemens, et al., 2021). Initially, both aimed to assess the efficacy of a two-dose regimen of the standard dose (SD/SD). However, a subgroup of participants received a lower priming dose of 2.2 x $10^{10}$ vp, followed by a standard dose of 5 x $10^{10}$ vp (LD/SD). While vaccine efficacy was calculated as 62% for SD/SD participants, it increased to 90% for LD/SD participants with a 4-week prime-boost interval. However, SD/SD with a longer interval (12 weeks or more) had a similarly high efficacy (VoyseyCosta Clemens, et al., 2021);this was the regimen approved by MHRA in December 2020. Reduced anti-vector immunity to the lower first dose along with waning anti-vector response over the 12-week interval have been suggested as potential explanations for the different efficacies of the tested regimens.

AstraZeneca received MHRA approval for this vaccine in December 2020, implementing a SD/SD regimen with a 12-week interval. The Joint Committee on Vaccination and Immunisation (JCVI) then recommended prioritising first doses of any vaccine, giving the second dose at up to 12 weeks (Mahase, 2020). Researchers have since updated the efficacy results, including data from all 4 clinical trials and some post-hoc exploratory analysis of the effect of extending the prime-boost interval (VoyseyCosta Clemens, et al., 2021) Efficacy and IgG and nAb titres were higher among those who received the booster at 12 weeks or later compared to those who received it within 6 weeks. Protection did not appear to significantly wane during the 12-week interval. This provides compelling evidence that the JCVI's recommendation was the correct response in a pandemic setting.

The efficacy of Gam-COVID-Vac in phase 3 was reported to be 91.6%, with 16 cases of COVID-19 among the 14,964 participants in the vaccine group compared to 62 cases among the 4,902 in the placebo group (Denis Y Logunov et al., 2021). Gam-COVID-Vac stands out among the viral vectored candidates as that with the highest efficacy reported thus far and the only candidate to use two human adenovirus serotypes (Ad26 and Ad5) in a heterologous prime-boost approach. AstraZeneca are now sponsoring a clinical trial to evaluate the immunogenicity of one dose of its vaccine in combination with one dose of Gam-COVID-Vac, with an estimated completion date of October 12, 2021 ("AstraZeneca Vax - Sputnik V Combination Study,").

The phase 3 study of the Janssen/Johnson & Johnson Ad26.COV2.S vaccine showed a single dose to be 66% effective in preventing moderate to severe COVID-19 28 days after vaccination, based on 468 symptomatic cases among 43,783 participants ("Johnson & Johnson Announces Single-Shot Janssen COVID-19 Vaccine Candidate Met Primary Endpoints in Interim Analysis of its Phase 3 ENSEMBLE Trial | Johnson & Johnson,"), it was on this basis that the EMA and the FDA granted emergency use authorisation for this candidate.

CanSino's vaccine candidate, based on Ad5, had 65.7% efficacy, as announced by press release ("China's CanSino Covid Vaccine Shows 65.7% Efficacy," 2021). It is unclear how many participants were included in efficacy analysis as data from phase 3 remain unpublished. Despite relatively lower efficacies of Ad5-nCoV and Ad26.COV2.S, their single-dose regimen may prove attractive to countries which face logistical problems in vaccine administration.

## ***SARS-CoV-2 Variants and Vaccine Efficacy***

The low fidelity of the RNA virus replicase enzymes normally results in the generation of diverse viral populations. However, coronaviruses possess a 3' exonuclease that can proof-read replicated genomes and reduce the frequency of errors. Despite this, several notable variants have emerged and become dominant: B.1.1.7, B.1.351 and P.1 and P.2. While all vaccines developed to date are derived from the Spike protein sequence containing the D614 residue, most circulating variants now contain the G614 residue. This substitution has been found to increase transmissibility with little impact on neutralisation. However, B.1.351, P.1 and P.2 variants have been found to contain several additional mutations in their Spike sequences that confer reduced susceptibility to neutralising antibodies (Wibmer et al., 2021).

Three of these mutations (N501Y, K417N and E484K) commonly occur together and are located within the RBD.

Several recent publications have assessed the ability of vaccine-induced responses to neutralise these variants. Wang et al. (2021) found a 1-3-fold decrease in the neutralisation sensitivity of pseudoviruses carrying combinations of variant residues by plasma taken from individuals given either of the approved mRNA vaccines. In comparison, the ability of convalescent plasma (CP), to neutralise variants was reduced by up to 29-fold, broadly in line with epidemiological reports of infections with the new strain in convalescent individuals. Madhi et al. (2021) found ChAdOx1 nCoV-19 to be ineffective at preventing mild to moderate disease caused by the B.1.351 variant, with 48% of serum samples unable to mediate neutralisation. Serum isolated from individuals vaccinated with Pfizer and ChAdOx was found have a reduction in their ability to neutralise the B.1.351 variant in vitro by 7.6 and 9-fold, respectively(Zhou et al., 2021).

The increasing global prevalence of these variants poses urgent questions on the efficacy of existing vaccines and is likely to require the development of new iterations of booster vaccines to ensure maximal coverage and protection against current and future variants. The minor changes required to create these new boosters could allow regulators to expedite their approval through use of existing safety profile data.

Efficacy against variant of SARS-CoV2, as recently highlighted by the Novavax news of its efficacy against the original strain 96.4% and the efficacy seen in South Africa 48.6% against Variant strains (Novavax 2021. www.novavax.com/covid-19-coronavirus-vaccine-candiate-updates.), suggests the same vaccine may be more effective in some areas that other, dependent on the circulating variants.

***Effectiveness of the vaccine***

Data from mass population vaccination efforts will provide valuable insights into the effectiveness of different vaccine candidates in different population groups. In a study of over 1 million Israeli citizens, BNT162b2 had an efficacy of 92% 7 days post-boost (Dagan et al., 2021), including individuals over 70 years of age. Real-world data is particularly helpful to assess the vaccines' ability to prevent disease, hospitalizations and transmission. In the UK, Public Health England calculated that those aged 70 or over who had been vaccinated with a single dose of BNT162b2 or ChAdOx1 nCoV-19 had their risk of emergency hospitalisation decreased by 43% and 37% respectively (Bernal et al., 2021). This was observed despite a reported decrease of immunogenicity demonstrated with BNT162b2 mRNA vaccine after the first dose in elderly populations (Abu Jabal et al., 2021). Overall, a recent study including 5.4 million adults in the UK showed an 85% and 94% reduction of COVID-19 related hospitalizations 28-34 days after a single dose of BNT162b2 and ChAdOx1, respectively (Vasileiou, 2021).

Early simulation experiments predicted that a vaccine efficacy of at least 60% was necessary across the whole population in order to stop the epidemic (Bartsch et al., 2020). However, several factors may effectively lower vaccination coverage including groups with co-morbidities and vaccine-hesitancy. Preliminary results from a safety and immune efficacy study showed that haematological cancer patients have a markedly decreased antibody response (<15%) to BNT162b2 compared to healthy individuals after the first dose (Monin-Aldama et al., 2021). In line with this, only 17% (76/436) of solid organ transplant recipients, who were vaccinated, developed antibodies at a median of 20 days after the first dose. Among the recipients who developed antibodies, 69% received mRNA-1273 and 32% received BNT162b2 (Boyarsky et al., 2021). This indicates that vaccination of individuals caring for vulnerable groups may be beneficial.

Furthermore, assessing effectiveness data worldwide allows researchers to monitor the effect of vaccines on emerging SARS-CoV-2 variants. For instance, the SIREN study demonstrated 72% effectiveness of BNT162b2 21 days after the first dose and 86% 7 days after the second dose in healthcare workers, despite the spread of B1.1.7 variant in the UK (Hall, 2021). However, the evidence on vaccines preventing the spread of new variants, viral shedding and asymptomatic infections is still too limited to justify removing preventative measures such as social distancing.

***Rapid response and remaining challenges***

The successful development of multiple SARS-CoV-2 vaccines has largely resulted from global funding investments and hitherto unprecedented international collaboration, allowing decades of research and know-how in vaccine development to come to fruition.

Clinical trial timelines have been compressed significantly, with regulatory bodies prioritising vaccine research and trials being carried out in parallel rather than sequentially. Furthermore, use of novel mRNA-based vaccines has accelerated vaccine development and delivery.

Data accumulated from the phase 3 clinical trials outlined above demonstrate high levels of efficacy at preventing COVID-19 across vaccine platforms. Disparities between dosage and booster intervals may account for moderate intra-platform variability in immunogenicity and reactogenicity. In addition, the lack of harmonisation in some immunological assays employed potentially explains the discrepancies in the reporting of immunogenicity data. This highlights the value of international standardisation in assessing SARS-CoV-2 immune markers, as is currently being advocated by the World Health Organization.

Longitudinal analysis of patient cohorts for infection, disease and retention of immune responses is ongoing and will provide insight into whether subsequent boosters are required for longer-term protection. Furthermore, recent emergence of SARS-CoV-2 variants could necessitate the prospective re-evaluation of vaccine-induced protection and development of modified vaccine boosters.

SARS-CoV-2 is likely to remain a public health problem for the foreseeable future, particularly with the added complications of vaccine hesitancy and refusal, and poor vaccine access in low- and middle-income countries. An argument can be made for more equitable distribution of vaccines to prevent evolutionary hotspots that will drive the emergence of new SARS-CoV-2 variants. It remains to be seen how the vaccine rollout will affect virus mutation rates and alter viral evolution globally. In general, however, the research infrastructure and collaborations established since the start of the pandemic will undoubtedly help reduce the burden of disease and accelerate the development of medical innovations more broadly.

***Box 1: What is the consensus on COVID-19 vaccine development?***

Despite significant advances in SARS-CoV-2 treatments, mass vaccination remains the most critical tool in countering the COVID-19 pandemic. There are twelve vaccine candidates approved for full or limited use worldwide, with many more currently being assessed in clinical and pre-clinical trials. These candidates are based on a variety of vaccine platforms including RNA, protein subunit, inactivated virus, and viral vectors. All these platforms are based on a strong foundation of years of research efforts in these technologies.

In April 2020, the WHO set out criteria for a viable vaccine candidate which included a minimum 50% efficacy against severe disease and a favourable safety profile. Candidates across a variety of platforms have met these criteria, including several vaccines which are estimated to have over 90% efficacy against severe disease, comparable to the most effective licensed vaccines to date. Candidates have proved to be safe, with mostly mild, time-limited side effects.

***Box 2: Remaining Unknowns in COVID-19 Vaccine development***

While all vaccines have been shown to be safe and effective, there are variations in reported efficacies due to the lack of standardisation of the trial protocols and defined end-points. A critical research question to be answered centres around the longevity of vaccine-induced protection. Waning immune response following vaccination may necessitate regular booster shots. It is currently proposed that booster shots may be required on a biennial basis, as seen for patients previously infected with SARS-CoV-1 and MERS, where immunity lasted 2-3 years (Poland, Ovsyannikova, & Kennedy, 2020). In addition, clinical trials thus far have only assessed protection against symptomatic disease and more information on prevention of asymptomatic transmission will be required.

As SARS-CoV-2 remains in circulation, mutations leading to increased transmissibility or virulence represent a growing challenge. Therefore, current research efforts focus on the efficacy against these emerging variants and modification of existing vaccine platforms to ensure appropriate protection.

**Figure 1. The four dominant SARS-CoV-2 vaccine platforms.** Inactivated and protein vaccines elicit a strong humoral response. in contrast, mRNA and viral vector vaccines elicit a stronger cellular immune response due to the production of vaccine antigens by host cells and cross-presentation on human leukocyte antigen class 1. Based on this endogenous production of viral antigens, mRNA and viral vector vaccines typically do not require addition of adjuvants. However, protein and inactivated vaccines still outcompete RNA and viral vector vaccines in terms of costs and stability.

Tables

Table 1. Inactivated Vaccines

| **Name** | **Manufacturer** | **Booster** | **Efficacy** |
|---|---|---|---|
| BBIBP-CorV | Sinopharm and the Wuhan Institute of Biological Products | Day 21 | 79.34% |
| CoronaVac | Sinovac Biotech | Day 28 | 50.4%* |

* Varying efficacy data have been provided by trials in Turkey, Indonesia, Brazil . (Mahase, 2021)
(Trial numbers: BBIBP-CorV NCT04352608 phase 1 / 2, NCT04560881 phase 3. CoronaVac NCT04383574 phase 1 / 2, NCT04582344 phase 3 Turkey, NCT04508075 PHASE 3 Indonesia.)

Table 2. RNA Vaccines

| **Name** | **Manufacturer** | **Booster** | **Efficacy** * |
|---|---|---|---|
| mRNA-1273 | Moderna in partnership with the National Institute of Allergies and Infectious Diseases (NIAID) | Day 28 | 94.5~~10~~% |
| BNT162b2 | BioNTech and Pfizer | Day 21 | 95% |

(Trial numbers: mRNA-1273 NCT04470427, BNT162b2 NCT04368728.)

Table 3. Protein Vaccines

| **Name** | **Manufacturer** | **Booster** | **Efficacy** 8* |
|---|---|---|---|
| NVX-CoV2373 | Novavax | Day 21 | 96.4% ** |
| ZF2001 | Anhui Zhifei Longcom in partnership with the Chinese Academy of Medical Sciences | Day 14 | Not calculated |

(Trial numbers: NVX-CoV2373 NCT04368988 phase 1 / 2, NCT04611802 phase 3. ZF2001, NCT04646590 nphase 3.) *(BMJ 2021;372:n597, http://dx.doi.org/10.1136/bmj.n597) **(Novavax, March11 2021.)

Table 4. Viral Vector Vaccines

| **Name** | **Manufacturer** | **Booster** | **Efficacy** *** |
|---|---|---|---|
| ChadOx1 nCoV-19 | AstraZeneca in partnership with the University of Oxford | Day 28-Day 84 * | 82.4%** |
| Gam-COVID-Vac (aka Sputnik V) | The Gamaleya Research Institute (Russian Ministry of Health) | Day 21 | 91.60% |

| | | | |
|---|---|---|---|
| Ad26.COV2.S | Johnson & Johnson | None | 72% |
| Ad5-nCoV | CanSino Biologics in partnership with the Academy of Military Medical Sciences | None | 65.7% |

*In clinical trials, booster doses were administered any time within 4 to 12 weeks of the initial dose. See below for further discussion of dosing intervals.

**The average efficacy across both SD/SD and LD/SD dosing regimens was originally calculated at 70.4% (VoyseyClemens, et al., 2021). On the inclusion of a further 5541 participants in the efficacy analysis, this was revised down (VoyseyCosta Clemens, et al., 2021). Data using 12 week boost showed 82.4% ***(BMJ 2021;372:n597, http://dx.doi.org/10.1136/bmj.n597)

(ChAdOx1 nCoV-19 NTC04444674 – South Africa, NTC04324606 – UK phase 1/2, NTC04400838 - UK phase 2/3, ADZ12222 – Phase 3 AstraZeneca. Gam-COVID-VacNCT04437875 phase 1 / 2, NCT04656613 phase 3. AD26.COV2.S NCT04436276 phase 1/2, NCT04505722 phase 3. Ad5-nCoV NCT04552366 phase 1, NTC04341389 phase 2, NCT04526990 phase 3.)

## ***Acronyms***

CP – Convalescent Plasma
EMA – European Medicines Agency
FDA – United States Food and Drug Administration
GMT – Geometric Mean Titre
IM – Intramuscular
JCVI - Joint Committee on Vaccination and Immunisation
MERS – Middle East Respiratory Syndrome coronavirus
MHRA- Medicines and Healthcare Products Regulatory Agency (UK)
nAbs – Neutralising Antibodies
RBD – Receptor Binding Domain
SARS – Severe Acute Respiratory Syndrome coronavirus
UTR – Untranslated Region
Vp – Viral particles
WHO – World Health Organisation

## Funding

*JK is funded by Vaccitech. JA is funded jointly by the Centre for Osteoarthritis Pathogenesis Versus Arthritis (Grant reference 21621) and the Centre for Sport, Exercise and Osteoarthritis Research Versus Arthritis (Grant reference 21595). VB is funded by a FOREUM grant and is a member of the Centre for OA Pathogenesis Versus Arthritis (21621). MO'H is funded by Cancer Research UK (Grant reference HJ2020629.) and is a member of the Centre for OA Pathogenesis Versus Arthritis (21621). LC is supported by a Oxford-Bristol Myers Squibb Fellowship. FR is funded by the Infection, Immunology and Translational Medicine Wellcome Trust Studentship*

## Acknowledgements

We wish to express our gratitude to all members of the Oxford-Cardiff COVID-19 Literature Consortium for their relentless support and hard work during this pandemic. We also thank Professor Anita for her helpful comments on this manuscript.

Authors' contribution

All named authors have contributed by reviewing the current peer-reviewed and preprint literature and writing of the manuscript. The consortium has provided a platform for a wide-range literature assessment in the context of local and joint COVID-19 literature initiatives and organised cross-university review efforts. All authors have approved the final version.

Conflict of interest: Jakub Kopycinski receives grant funding from VacciTech.

Conflict of interest: Jennifer Alderson assisted with ChAdOx1 nCoV 19 trial, but did not take part in writing about this vaccine.

## References

Abu Jabal, K., Ben-Amram, H., Beiruti, K., Batheesh, Y., Sussan, C., Zarka, S., & Edelstein, M. (2021). Impact of age, ethnicity, sex and prior infection status on immunogenicity following a single dose of the BNT162b2 mRNA COVID-19 vaccine: real-world evidence from healthcare workers, Israel, December 2020 to January 2021. *Eurosurveillance, 26*(6), 2100096. doi:doi:https://doi.org/10.2807/1560-7917.ES.2021.26.6.2100096

Arya, R., Kumari, S., Pandey, B., Mistry, H., Bihani, S. C., Das, A., . . . Kumar, M. (2021). Structural insights into SARS-CoV-2 proteins. *Journal of Molecular Biology, 433*(2), 166725. doi:https://doi.org/10.1016/j.jmb.2020.11.024

AstraZeneca Vax - Sputnik V Combination Study. In: https://ClinicalTrials.gov/show/NCT04684446.

Baden, L. R., El Sahly, H. M., Essink, B., Kotloff, K., Frey, S., Novak, R., . . . Zaks, T. (2020). Efficacy and Safety of the mRNA-1273 SARS-CoV-2 Vaccine. *New England Journal of Medicine, 384*(5), 403-416. doi:10.1056/NEJMoa2035389

Bartsch, S. M., O'Shea, K. J., Ferguson, M. C., Bottazzi, M. E., Wedlock, P. T., Strych, U., . . . Lee, B. Y. (2020). Vaccine Efficacy Needed for a COVID-19 Coronavirus Vaccine to Prevent or Stop an Epidemic as the Sole Intervention. *American Journal of Preventive Medicine, 59*(4), 493-503. doi:10.1016/j.amepre.2020.06.011

Bernal, J. L., Andrews, N., Gower, C., Stowe, J., Robertson, C., Tessier, E., . . . Ramsay, M. (2021). Early effectiveness of COVID-19 vaccination with BNT162b2 mRNA vaccine and ChAdOx1 adenovirus vector vaccine on symptomatic disease, hospitalisations and mortality in older adults in England. *medRxiv*, 2021.2003.2001.21252652. doi:10.1101/2021.03.01.21252652

Boyarsky, B. J., Werbel, W. A., Avery, R. K., Tobian, A. A. R., Massie, A. B., Segev, D. L., & Garonzik-Wang, J. M. (2021). Immunogenicity of a Single Dose of SARS-CoV-2 Messenger RNA Vaccine in Solid Organ Transplant Recipients. *JAMA*. doi:10.1001/jama.2021.4385

China's CanSino Covid Vaccine Shows 65.7% Efficacy. (2021, 2021-02-08T12:46:52.703Z, 2021-02-08T12:46:52.703Z, 2021-02-08T12:46:52.703Z, 2021-03-12T20:24:17.315Z, 2021-03-12T20:24:17.315Z, 2021-03-12T20:24:17.315Z). *Bloomberg.com*.

Dagan, N., Barda, N., Kepten, E., Miron, O., Perchik, S., Katz, M. A., . . . Balicer, R. D. (2021). BNT162b2 mRNA Covid-19 Vaccine in a Nationwide Mass Vaccination Setting. *New England Journal of Medicine*. doi:10.1056/NEJMoa2101765

Ewer, K. J., Barrett, J. R., Belij-Rammerstorfer, S., Sharpe, H., Makinson, R., Morter, R., . . . Lambe, T. (2020). T cell and antibody responses induced by a single dose of ChAdOx1 nCoV-19 (AZD1222) vaccine in a phase 1/2 clinical trial. *Nature Medicine*, 1-9. doi:10.1038/s41591-020-01194-5

Folegatti, P. M., Ewer, K. J., Aley, P. K., Angus, B., Becker, S., Belij-Rammerstorfer, S., . . . Yau, Y. (2020). Safety and immunogenicity of the ChAdOx1 nCoV-19 vaccine against SARS-CoV-2: a preliminary report of a phase 1/2, single-blind, randomised controlled trial. *The Lancet, 396*(10249), 467-478. doi:10.1016/S0140-6736(20)31604-4

Fries, L., Cho, I., Krahling, V., Fehling, S. K., Strecker, T., Becker, S., . . . Glenn, G. (2020). Randomized, Blinded, Dose-Ranging Trial of an Ebola Virus Glycoprotein Nanoparticle Vaccine With Matrix-M Adjuvant in Healthy Adults. *J Infect Dis, 222*(4), 572-582. doi:10.1093/infdis/jiz518

Hall, V. J., Foulkes, S., Saei, A., Andrews, N., Oguti, B., Charlett, A., Wellington, E., Stowe, J., Gillson, N., Atti, A., Islam J., Karagiannis, I., Munro, K., Khawam, J., The SIREN Study Group, Chand, M. A., Brown, C., Ramsay, M. E., Bernal, J. L., Hopkins, S. (2021). Effectiveness of BNT162b2 mRNA Vaccine Against Infection and COVID-19 Vaccine Coverage in Healthcare Workers in England, Multicentre Prospective Cohort Study (the SIREN Study). *Preprints with THE LANCET*. doi:http://dx.doi.org/10.2139/ssrn.3790399

Harvey, R. A., Rassen, J. A., Kabelac, C. A., Turenne, W., Leonard, S., Klesh, R., . . . Penberthy, L. T. (2021). Association of SARS-CoV-2 Seropositive Antibody Test With Risk of Future Infection. *JAMA Internal Medicine*. doi:10.1001/jamainternmed.2021.0366

Henao-Restrepo, A. M., Longini, I. M., Egger, M., Dean, N. E., Edmunds, W. J., Camacho, A., . . . Røttingen, J.-A. (2015). Efficacy and effectiveness of an rVSV-vectored vaccine expressing Ebola surface glycoprotein: interim results from the Guinea ring vaccination cluster-randomised trial. *The Lancet, 386*(9996), 857-866. doi:10.1016/S0140-6736(15)61117-5

Huang, Y., Yang, C., Xu, X.-f., Xu, W., & Liu, S.-w. (2020). Structural and functional properties of SARS-CoV-2 spike protein: potential antivirus drug development for COVID-19. *Acta Pharmacologica Sinica, 41*(9), 1141-1149. doi:10.1038/s41401-020-0485-4

Jackson, L. A., Anderson, E. J., Rouphael, N. G., Roberts, P. C., Makhene, M., Coler, R. N., . . . Beigel, J. H. (2020). An mRNA Vaccine against SARS-CoV-2 — Preliminary Report. *New England Journal of Medicine, 383*(20), 1920-1931. doi:10.1056/NEJMoa2022483

Johnson & Johnson Announces Single-Shot Janssen COVID-19 Vaccine Candidate Met Primary Endpoints in Interim Analysis of its Phase 3 ENSEMBLE Trial | Johnson & Johnson. *Content Lab U.S.*

Keech, C., Albert, G., Cho, I., Robertson, A., Reed, P., Neal, S., . . . Glenn, G. M. (2020). Phase 1-2 Trial of a SARS-CoV-2 Recombinant Spike Protein Nanoparticle Vaccine. *N Engl J Med*. doi:10.1056/NEJMoa2026920

Keech, C., Albert, G., Reed, P., Neal, S., Plested, J. S., Zhu, M., . . . Glenn, G. M. (2020). First-in-Human Trial of a SARS CoV 2 Recombinant Spike Protein Nanoparticle Vaccine. *medRxiv*, 2020.2008.2005.20168435. doi:10.1101/2020.08.05.20168435

Killikelly, A. M., Kanekiyo, M., & Graham, B. S. (2016). Pre-fusion F is absent on the surface of formalin-inactivated respiratory syncytial virus. *Scientific Reports, 6*(1), 34108. doi:10.1038/srep34108

Kremsner, P., Mann, P., Bosch, J., Fendel, R., Gabor, J. J., Kreidenweiss, A., . . . Oostvogels, L. (2020). Phase 1 Assessment of the Safety and Immunogenicity of an mRNA- Lipid Nanoparticle Vaccine Candidate Against SARS-CoV-2 in Human Volunteers. *medRxiv*, 2020.2011.2009.20228551. doi:10.1101/2020.11.09.20228551

Libster, R., Pérez Marc, G., Wappner, D., Coviello, S., Bianchi, A., Braem, V., . . . Polack, F. P. (2021). Early High-Titer Plasma Therapy to Prevent Severe Covid-19 in Older Adults. *New England Journal of Medicine, 384*(7), 610-618. doi:10.1056/NEJMoa2033700

Logunov, D. Y., Dolzhikova, I. V., Shcheblyakov, D. V., Tukhvatulin, A. I., Zubkova, O. V., Dzharullaeva, A. S., . . . Gintsburg, A. L. (2021). Safety and efficacy of an rAd26 and rAd5 vector-based heterologous prime-boost COVID-19 vaccine: an interim analysis of a randomised controlled phase 3 trial in Russia. *The Lancet, 397*(10275), 671-681. doi:10.1016/S0140-6736(21)00234-8

Logunov, D. Y., Dolzhikova, I. V., Zubkova, O. V., Tukhvatulin, A. I., Shcheblyakov, D. V., Dzharullaeva, A. S., . . . Gintsburg, A. L. (2020). Safety and immunogenicity of an rAd26 and rAd5 vector-based heterologous prime-boost COVID-19 vaccine in two formulations: two open, non-randomised phase 1/2 studies from Russia. *The Lancet, 396*(10255), 887-897. doi:10.1016/S0140-6736(20)31866-3

Madhi, S. A., Baillie, V., Cutland, C. L., Voysey, M., Koen, A. L., Fairlie, L., . . . Izu, A. (2021). Safety and efficacy of the ChAdOx1 nCoV-19 (AZD1222) Covid-19 vaccine against the B.1.351 variant in South Africa. *medRxiv*, 2021.2002.2010.21251247. doi:10.1101/2021.02.10.21251247

Mahase, E. (2020). Covid-19: Order to reschedule and delay second vaccine dose is "totally unfair," says BMA. *BMJ, 371*, m4978. doi:10.1136/bmj.m4978

Mallapaty, S. (2021, 15 January 2021). China COVID vaccine reports mixed results — what does that mean for the pandemic? Retrieved from https://www.nature.com/articles/d41586-021-00094-z

Moderna Announces it has Shipped Variant-Specific Vaccine Candidate, mRNA-1273.351, to NIH for Clinical Study | Moderna, Inc.

Monin-Aldama, L., Laing, A. G., Muñoz-Ruiz, M., McKenzie, D. R., del Molino del Barrio, I., Alaguthurai, T., . . . Irshad, S. (2021). Interim results of the safety and immune-efficacy of 1

versus 2 doses of COVID-19 vaccine BNT162b2 for cancer patients in the context of the UK vaccine priority guidelines. *medRxiv*, 2021.2003.2017.21253131. doi:10.1101/2021.03.17.21253131

Müller, L., Andrée, M., Moskorz, W., Drexler, I., Walotka, L., Grothmann, R., . . . Schaal, H. (2021). Age-dependent immune response to the Biontech/Pfizer BNT162b2 COVID-19 vaccination. *medRxiv*, 2021.2003.2003.21251066. doi:10.1101/2021.03.03.21251066

Mulligan, M. J., Lyke, K. E., Kitchin, N., Absalon, J., Gurtman, A., Lockhart, S., . . . Jansen, K. U. (2020). Phase I/II study of COVID-19 RNA vaccine BNT162b1 in adults. *Nature, 586*(7830), 589-593. doi:10.1038/s41586-020-2639-4

Nascimento, I. P., & Leite, L. C. C. (2012). Recombinant vaccines and the development of new vaccine strategies. *Brazilian journal of medical and biological research, 45*(12), 1102-1111. doi:10.1590/s0100-879x2012007500142

Novavax, I. (2021). Novavax Confirms High Levels of Efficacy Against Original and Variant COVID-19 Strains in United Kingdom and South Africa Trials [Press release]. Retrieved from https://ir.novavax.com/news-releases/news-release-details/novavax-confirms-high-levels-efficacy-against-original-and-0

Palacios, R., Patiño, E. G., de Oliveira Piorelli, R., Conde, M. T. R. P., Batista, A. P., Zeng, G., . . . Gast, C. (2020). Double-Blind, Randomized, Placebo-Controlled Phase III Clinical Trial to Evaluate the Efficacy and Safety of treating Healthcare Professionals with the Adsorbed COVID-19 (Inactivated) Vaccine Manufactured by Sinovac – PROFISCOV: A structured summary of a study protocol for a randomised controlled trial. *Trials, 21*(1), 853. doi:10.1186/s13063-020-04775-4

Pardi, N., Hogan, M. J., Porter, F. W., & Weissman, D. (2018). mRNA vaccines — a new era in vaccinology. *Nature Reviews Drug Discovery, 17*(4), 261-279. doi:10.1038/nrd.2017.243

A Phase III Clinical Trial to Determine the Safety and Efficacy of ZF2001 for Prevention of COVID-19. In: https://ClinicalTrials.gov/show/NCT04646590.

Polack, F. P., Thomas, S. J., Kitchin, N., Absalon, J., Gurtman, A., Lockhart, S., . . . Gruber, W. C. (2020). Safety and Efficacy of the BNT162b2 mRNA Covid-19 Vaccine. *New England Journal of Medicine, 383*(27), 2603-2615. doi:10.1056/NEJMoa2034577

Poland, G. A., Ovsyannikova, I. G., & Kennedy, R. B. (2020). SARS-CoV-2 immunity: review and applications to phase 3 vaccine candidates. *The Lancet, 396*(10262), 1595-1606. doi:10.1016/S0140-6736(20)32137-1

Sadoff, J., Gars, M. L., Shukarev, G., Heerwegh, D., Truyers, C., Groot, A. M. d., . . . Schuitemaker, H. (2020). Safety and immunogenicity of the Ad26.COV2.S COVID-19 vaccine candidate: interim results of a phase 1/2a, double-blind, randomized, placebo-controlled trial. *medRxiv*, 2020.2009.2023.20199604. doi:10.1101/2020.09.23.20199604

Sahin, U., Muik, A., Derhovanessian, E., Vogler, I., Kranz, L. M., Vormehr, M., . . . Türeci, Ö. (2020). COVID-19 vaccine BNT162b1 elicits human antibody and TH1 T cell responses. *Nature, 586*(7830), 594-599. doi:10.1038/s41586-020-2814-7

A Study Looking at the Effectiveness, Immune Response, and Safety of a COVID-19 Vaccine in Adults in the United Kingdom. In: https://ClinicalTrials.gov/show/NCT04583995.

Tatsis, N., & Ertl, H. C. J. (2004). Adenoviruses as vaccine vectors. *Molecular Therapy, 10*(4), 616-629. doi:10.1016/j.ymthe.2004.07.013

Teijaro, J. R., & Farber, D. L. (2021). COVID-19 vaccines: modes of immune activation and future challenges. *Nature Reviews Immunology*. doi:10.1038/s41577-021-00526-x

Tian, J.-H., Patel, N., Haupt, R., Zhou, H., Weston, S., Hammond, H., . . . Smith, G. (2020). SARS-CoV-2 spike glycoprotein vaccine candidate NVX-CoV2373 elicits immunogenicity in baboons and protection in mice. *bioRxiv*, 2020.2006.2029.178509. doi:10.1101/2020.06.29.178509

Vartak, A., & Sucheck, S. J. (2016). Recent Advances in Subunit Vaccine Carriers. *Vaccines, 4*(2), 12. Retrieved from https://www.mdpi.com/2076-393X/4/2/12

Vasileiou, E., Bradley, D., Chuter, A., de Lusignan, S., Docherty, A., Ford, D., Hobbs, R., Joy, M., Katikireddi, S. V., Marple, J., McCowan, C., Simpson, C. R., McGagh, D., McMenamin, J., Moore, E., Murray, J-L. K., Pan, J., Ritchie, L., Shah, S. A., ... Beggs, J. (2021). Effectiveness of First Dose of COVID-19 Vaccines Against Hospital Admissions in Scotland: National Prospective Cohort Study of 5.4 Million People. . *The Lancet*. doi: https://ssrn.com/abstract=3789264

Voysey, M., Clemens, S. A. C., Madhi, S. A., Weckx, L. Y., Folegatti, P. M., Aley, P. K., . . . Zuidewind, P. (2021). Safety and efficacy of the ChAdOx1 nCoV-19 vaccine (AZD1222) against SARS-CoV-2: an interim analysis of four randomised controlled trials in Brazil, South Africa, and the UK. *The Lancet, 397*(10269), 99-111. doi:10.1016/S0140-6736(20)32661-1

Voysey, M., Costa Clemens, S. A., Madhi, S. A., Weckx, L. Y., Folegatti, P. M., Aley, P. K., . . . Zuidewind, P. (2021). Single-dose administration and the influence of the timing of the booster dose on immunogenicity and efficacy of ChAdOx1 nCoV-19 (AZD1222) vaccine: a pooled analysis of four randomised trials. *The Lancet, 397*(10277), 881-891. doi:10.1016/S0140-6736(21)00432-3

Walls, A. C., Park, Y.-J., Tortorici, M. A., Wall, A., McGuire, A. T., & Veesler, D. (2020). Structure, Function, and Antigenicity of the SARS-CoV-2 Spike Glycoprotein. *Cell, 181*(2), 281-292.e286. doi:https://doi.org/10.1016/j.cell.2020.02.058

Walsh, E. E., Frenck, R. W., Falsey, A. R., Kitchin, N., Absalon, J., Gurtman, A., . . . Gruber, W. C. (2020). Safety and Immunogenicity of Two RNA-Based Covid-19 Vaccine Candidates. *New England Journal of Medicine, 383*(25), 2439-2450. doi:10.1056/NEJMoa2027906

Wang, Z., Schmidt, F., Weisblum, Y., Muecksch, F., Barnes, C. O., Finkin, S., . . . Nussenzweig, M. C. (2021). mRNA vaccine-elicited antibodies to SARS-CoV-2 and circulating variants. *Nature*. doi:10.1038/s41586-021-03324-6

Wibmer, C. K., Ayres, F., Hermanus, T., Madzivhandila, M., Kgagudi, P., Oosthuysen, B., . . . Moore, P. L. (2021). SARS-CoV-2 501Y.V2 escapes neutralization by South African COVID-19 donor plasma. *bioRxiv*, 2021.2001.2018.427166. doi:10.1101/2021.01.18.427166

Xia, S., Zhang, Y., Wang, Y., Wang, H., Yang, Y., Gao, G. F., . . . Yang, X. (2021). Safety and immunogenicity of an inactivated SARS-CoV-2 vaccine, BBIBP-CorV: a randomised, double-blind, placebo-controlled, phase 1/2 trial. *The Lancet Infectious Diseases, 21*(1), 39-51. doi:10.1016/S1473-3099(20)30831-8

Yang, S., Li, Y., Dai, L., Wang, J., He, P., Li, C., . . . Gao, G. F. (2020). Safety and immunogenicity of a recombinant tandem-repeat dimeric RBD protein vaccine against COVID-19 in adults: pooled analysis of two randomized, double-blind, placebo-controlled, phase 1 and 2 trials. *medRxiv*, 2020.2012.2020.20248602. doi:10.1101/2020.12.20.20248602

Zhang, Y., Zeng, G., Pan, H., Li, C., Hu, Y., Chu, K., . . . Zhu, F. (2021). Safety, tolerability, and immunogenicity of an inactivated SARS-CoV-2 vaccine in healthy adults aged 18-59 years: a randomised, double-blind, placebo-controlled, phase 1/2 clinical trial. *The Lancet Infectious Diseases, 21*(2), 181-192. doi:10.1016/S1473-3099(20)30843-4

Zhou, D., Dejnirattisai, W., Supasa, P., Liu, C., Mentzer, A. J., Ginn, H. M., . . . Screaton, G. R. (2021). Evidence of escape of SARS-CoV-2 variant B.1.351 from natural and vaccine-induced sera. *Cell*. doi:https://doi.org/10.1016/j.cell.2021.02.037

Zhu, F.-C., Guan, X.-H., Li, Y.-H., Huang, J.-Y., Jiang, T., Hou, L.-H., . . . Chen, W. (2020). Immunogenicity and safety of a recombinant adenovirus type-5-vectored COVID-19 vaccine in healthy adults aged 18 years or older: a randomised, double-blind, placebo-controlled, phase 2 trial. *The Lancet, 396*(10249), 479-488. doi:10.1016/S0140-6736(20)31605-6

Zhu, F.-C., Li, Y.-H., Guan, X.-H., Hou, L.-H., Wang, W.-J., Li, J.-X., . . . Chen, W. (2020). Safety, tolerability, and immunogenicity of a recombinant adenovirus type-5 vectored COVID-19 vaccine: a dose-escalation, open-label, non-randomised, first-in-human trial. *The Lancet, 395*(10240), 1845-1854. doi:10.1016/S0140-6736(20)31208-3

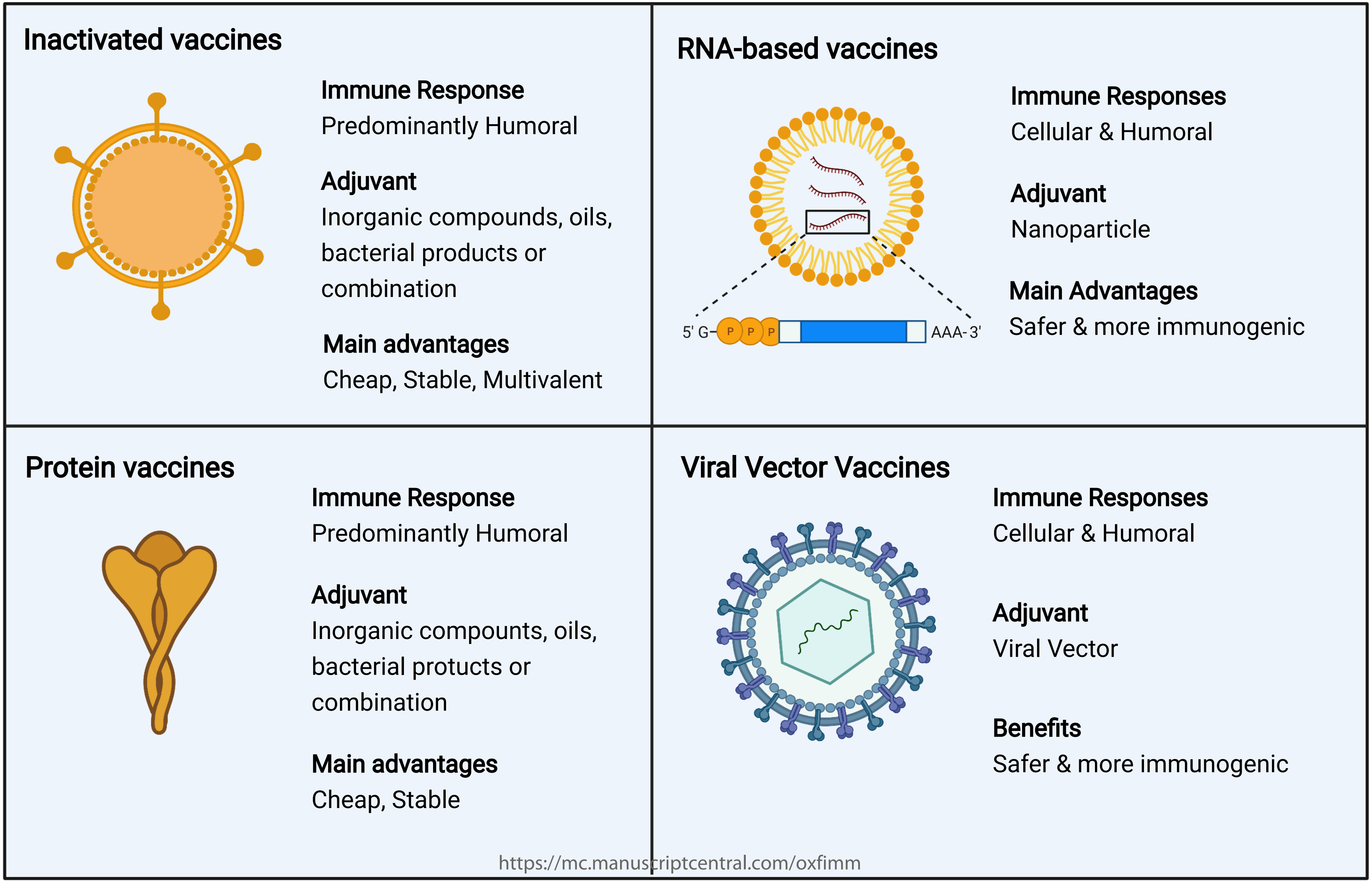

Inactivated vaccines
Immune Response
Predominantly Humoral
Adjuvant
Inorganic compounds, oils, bacterial products or combination
Main advantages
Cheap, Stable, Multivalent
RNA-based vaccines
5' G-
P
P
P
AAA-3'
Immune Responses
Cellular & Humoral
Adjuvant
Nanoparticle
Main Advantages
Safer & more immunogenic
Protein vaccines
Immune Response
Predominantly Humoral
Adjuvant
Inorganic compounds, oils, bacterial protucts or combination
Main advantages
Cheap, Stable
Viral Vector Vaccines
Immune Responses
Cellular & Humoral
Adjuvant
Viral Vector
Benefits
Safer & more immunogenic
